# Supplementary material for: Association between childhood maltreatment, psychopathology and DNA methylation of genes involved in stress regulation: Evidence from a study in Borderline Personality Disorder
Source: PLoS One. 2021 Mar 11;16(3):e0248514. doi: 10.1371/journal.pone.0248514 (PMC7951851; doi:10.1371/journal.pone.0248514)
Supplement: S1 Table — Group differences were calculated by Mann-Whitney-U tests (U, p); significant differences are marked in bold. These differences would did not survive correction due to multiple testing. Bonferroni correction for p = 0.05/42 = 0.0012. (DOCX) [file pone.0248514.s001.docx]

**S1 Table. Means (*M*) and standard deviations (*SD*) of NR3C1(exon 1*_F_*) methylation levels in absolute (%), mean-centered and ranked data for patients with BPD (n = 45) and HC (n = 44). Group differences were calculated by Mann-Whitney-U tests (*U, p*); significant differences are marked in bold. These differences did not survive correction for multiple testing.**

| **CpG** | **absolute *M* in %** | | **absolute *SD*** | | **mean-centered *M*** | | **Mean-centered *SD*** | | **Mean rank** | | **Mann-Whitney-*U*-test** | |
| --- | --- | --- | --- | --- | --- | --- | --- | --- | --- | --- | --- | --- |
|  | **BPD** | **HC** | **BPD** | **HC** | **BPD** | **HC** | **BPD** | **HC** | **BPD** | **HC** | ***U*** | ***p*** |
|  |  |  |  |  |  |  |  |  |  |  |  |  |
| 1 | 0.667 | 0.682 | 0.798 | 0.708 | -0.010 | 0.010 | 1.063 | 0.943 | 44.13 | 45.89 | 951.0 | 0.723 |
| 2 | 0.556 | 0.705 | 0.624 | 0.930 | -0.093 | 0.095 | 0.791 | 1.178 | 43.77 | 46.26 | 934.5 | 0.612 |
| 3 | 0.733 | 1.114 | 0.751 | 1.039 | -0.204 | 0.209 | 0.816 | 1.130 | 40.46 | 49.65 | 785.5 | 0.072 |
| 4 | 1.044 | 1.227 | 0.737 | 1.075 | -0.098 | 0.101 | 0.802 | 1.170 | 43.44 | 46.59 | 920.0 | 0.521 |
| 5 | 0.533 | 0.682 | 0.726 | 0.639 | -0.107 | 0.110 | 1.061 | 0.933 | 41.64 | 48.43 | 839.0 | 0.168 |
| 6 | 0.822 | 0.909 | 0.806 | 0.910 | -0.050 | 0.051 | 0.942 | 1.064 | 44.19 | 45.83 | 953.5 | 0.748 |
| 7 | 0.333 | 0.409 | 0.640 | 0.658 | -0.058 | 0.059 | 0.990 | 1.019 | 43.26 | 46.78 | 911.5 | 0.418 |
| 8 | 0.467 | 0.545 | 0.548 | 0.663 | -0.064 | 0.066 | 0.905 | 1.095 | 44.11 | 45.91 | 950.0 | 0.709 |
| 9 | 0.689 | 0.841 | 0.701 | 0.645 | -0.111 | 0.114 | 1.040 | 0.956 | 41.98 | 48.09 | 854.0 | 0.206 |
| 10 | 0.489 | 0.500 | 0.626 | 0.821 | -0.008 | 0.008 | 0.864 | 1.133 | 46.17 | 43.81 | 937.5 | 0.616 |
| **11** | 0.356 | 0.614 | 0.679 | 0.579 | -0.199 | 0.203 | 1.059 | 0.903 | 38.96 | 51.18 | 718.0 | **0.010** |
| 12 | 0.689 | 0.886 | 0.668 | 0.784 | -0.134 | 0.137 | 0.915 | 1.073 | 42.03 | 48.03 | 856.5 | 0.228 |
| 13 | 0.489 | 0.614 | 0.661 | 0.813 | -0.083 | 0.085 | 0.895 | 1.101 | 43.42 | 46.61 | 919.0 | 0.509 |
| 14 | 0.533 | 0.568 | 0.588 | 0.545 | -0.031 | 0.031 | 1.042 | 0.967 | 44.04 | 45.98 | 947.0 | 0.688 |
| 15 | 0.556 | 0.455 | 1.013 | 0.663 | 0.058 | -0.060 | 1.185 | 0.776 | 45.58 | 44.41 | 964.0 | 0.804 |
| 16 | 0.444 | 0.705 | 0.624 | 0.851 | -0.171 | 0.175 | 0.829 | 1.132 | 41.53 | 48.55 | 834.0 | 0.149 |
| 17 | 0.667 | 0.886 | 0.707 | 0.841 | -0.139 | 0.142 | 0.907 | 1.079 | 42.10 | 47.97 | 859.5 | 0.243 |
| 18 | 1.089 | 0.841 | 1.690 | 0.861 | 0.091 | -0.093 | 1.258 | 0.641 | 45.88 | 44.10 | 950.5 | 0.720 |
| 19 | 0.622 | 0.909 | 0.684 | 0.741 | -0.196 | 0.201 | 0.945 | 1.025 | 40.38 | 49.73 | 782.0 | 0.060 |
| 20 | 0.444 | 0.523 | 0.755 | 0.590 | -0.057 | 0.059 | 1.117 | 0.873 | 42.24 | 47.82 | 866.0 | 0.239 |
| 21 | 0.511 | 0.636 | 0.626 | 0.487 | -0.110 | 0.113 | 1.114 | 0.866 | 41.71 | 48.36 | 842.0 | 0.167 |
| 22 | 0.489 | 0.545 | 0.626 | 0.697 | -0.042 | 0.043 | 0.950 | 1.058 | 44.29 | 45.73 | 958.0 | 0.765 |
| **23** | 0.267 | 0.545 | 0.495 | 0.663 | -0.231 | 0.236 | 0.829 | 1.109 | 39.86 | 50.26 | 758.5 | **0.023** |
| 24 | 0.622 | 0.818 | 0.747 | 1.018 | -0.109 | 0.111 | 0.838 | 1.141 | 42.90 | 47.15 | 895.5 | 0.393 |
| 25 | 0.489 | 0.773 | 0.589 | 0.912 | -0.181 | 0.185 | 0.760 | 1.177 | 41.22 | 48.86 | 820.0 | 0.119 |
| 26 | 0.867 | 0.909 | 1.307 | 0.802 | -0.019 | 0.020 | 1.209 | 0.741 | 42.00 | 48.07 | 855.0 | 0.232 |
| 27 | 0.311 | 0.409 | 0.633 | 0.583 | -0.080 | 0.082 | 1.042 | 0.960 | 42.51 | 47.55 | 878.0 | 0.252 |
| 28 | 0.244 | 0.432 | 0.484 | 0.587 | -0.171 | 0.175 | 0.893 | 1.081 | 41.36 | 48.73 | 826.0 | 0.093 |
| 29 | 0.333 | 0.477 | 0.564 | 0.792 | -0.104 | 0.106 | 0.822 | 1.154 | 43.26 | 46.78 | 911.5 | 0.433 |
| **30** | 0.400 | 0.795 | 0.618 | 0.734 | -0.278 | 0.285 | 0.879 | 1.045 | 38.40 | 51.75 | 693.0 | **0.007** |
| 31 | 0.622 | 0.614 | 1.029 | 0.754 | 0.005 | -0.005 | 1.145 | 0.839 | 44.12 | 45.90 | 950.5 | 0.716 |
| 32 | 0.289 | 0.318 | 0.549 | 0.561 | -0.026 | 0.027 | 0.994 | 1.017 | 44.40 | 45.61 | 963.0 | 0.772 |
| 33 | 0.444 | 0.455 | 0.624 | 0.548 | -0.009 | 0.009 | 1.068 | 0.938 | 44.26 | 45.76 | 956.5 | 0.750 |
| 34 | 0.200 | 0.341 | 0.405 | 0.526 | -0.148 | 0.151 | 0.859 | 1.116 | 42.30 | 47.76 | 868.5 | 0.189 |
| 35 | 0.422 | 0.295 | 0.621 | 0.553 | 0.106 | -0.109 | 1.055 | 0.940 | 47.33 | 42.61 | 885.0 | 0.284 |
| 36 | 0.644 | 0.750 | 0.645 | 0.615 | -0.083 | 0.085 | 1.026 | 0.977 | 42.90 | 47.15 | 895.5 | 0.386 |
| 37 | 0.333 | 0.591 | 0.674 | 0.923 | -0.157 | 0.160 | 0.830 | 1.136 | 41.42 | 48.66 | 829.0 | 0.109 |
| 38 | 0.489 | 0.659 | 0.626 | 0.939 | -0.106 | 0.108 | 0.786 | 1.179 | 43.62 | 46.41 | 928.0 | 0.564 |
| 39 | 0.311 | 0.432 | 0.468 | 0.545 | -0.117 | 0.120 | 0.921 | 1.073 | 42.69 | 47.36 | 886.0 | 0.306 |
| 40 | 0.822 | 0.500 | 1.051 | 0.629 | 0.181 | 1.196 | -0.185 | 0.716 | 48.50 | 41.42 | 832.5 | 0.150 |
| 41 | 0.867 | 0.841 | 0.968 | 0.861 | 0.014 | -0.014 | 1.062 | 0.945 | 44.99 | 45.01 | 989.5 | 0.996 |
| 42 | 0.422 | 0.614 | 0.543 | 0.689 | -0.152 | 0.155 | 0.871 | 1.105 | 41.94 | 48.13 | 852.5 | 0.199 |
| **mean** | 0.658 | 0.739 | 0.144 | 0.169 | -0.083 | 0.085 | 0.218 | 0.295 | 37.87 | 52.30 | 669.0 | **0.008*** |

Bonferroni correction for *p* = 0.05/42 = 0.0012.
