# Supplementary material for: Association between childhood maltreatment, psychopathology and DNA methylation of genes involved in stress regulation: Evidence from a study in Borderline Personality Disorder
Source: PLoS One. 2021 Mar 11;16(3):e0248514. doi: 10.1371/journal.pone.0248514 (PMC7951851; doi:10.1371/journal.pone.0248514)
Supplement: S2 Table — Group differences were calculated by Mann-Whitney-U tests (U, p). (DOCX) [file pone.0248514.s002.docx]

**S2 Table. Means (*M*) and standard deviations (*SD*) of FKBP5 methylation levels in absolute, mean-centered and ranked data for patients with BPD (n = 44) and HC (n = 44). Group differences were calculated by Mann-Whitney-U tests (*U, p*).**

| **CpG** | **absolute *M* in %** | | **absolute *SD*** | | **mean-centered *M*** | | **Mean-centered SD** | | **Mean rank** | | **Mann-Whitney-*U*-test** | |
| --- | --- | --- | --- | --- | --- | --- | --- | --- | --- | --- | --- | --- |
|  | **BPD** | **HC** | **BPD** | **HC** | **BPD** | **HC** | **BPD** | **HC** | **BPD** | **HC** | ***U*** | ***p*** |
| bin 1_1 | 86.386 | 86.296 | 3.237 | 3.085 | 0.0145 | -0.014 | 1.030 | 0.981 | 45.06 | 43.94 | 943.5 | 0.837 |
| bin 1_2 | 74.341 | 74.682 | 5.256 | 3.542 | -0.038 | 0.038 | 1.179 | 0.794 | 47.30 | 41.70 | 845.0 | 0.302 |
| bin 2_1 | 95.909 | 95.273 | 1.491 | 1.897 | 0.184 | -0.184 | 0.864 | 1.099 | 48.17 | 40.83 | 806.5 | 0.169 |
| bin 2_2 | 93.000 | 92.068 | 2.770 | 3.202 | 0.155 | -0.155 | 0.919 | 1.063 | 48.31 | 40.69 | 800.5 | 0.159 |
| bin 2_3 | 72.227 | 70.955 | 4.170 | 4.559 | 0.145 | -0.145 | 0.950 | 1.038 | 48.03 | 40.97 | 812.5 | 0.193 |
| bin 1 mean | 80.364 | 80.489 | 3.466 | 2.596 | -0.012 | 0.012 | 0.889 | 0.697 | 45.36 | 43.64 | 930.0 | 0.751 |
| bin 2 mean | 87.046 | 86.099 | 2.427 | 2.826 | 0.161 | -0.161 | 0.777 | 0.926 | 48.80 | 40.20 | 779.0 | 0.115 |
| mean | 84.375 | 83.857 | 2.478 | 2.456 | 0.092 | -0.092 | 0.728 | 0.756 | 47.41 | 41.59 | 840.0 | 0.285 |
|  |  |  |  |  |  |  |  |  |  |  |  |  |
