# Supplementary material for: Association between childhood maltreatment, psychopathology and DNA methylation of genes involved in stress regulation: Evidence from a study in Borderline Personality Disorder
Source: PLoS One. 2021 Mar 11;16(3):e0248514. doi: 10.1371/journal.pone.0248514 (PMC7951851; doi:10.1371/journal.pone.0248514)
Supplement: S3 Table — Bonferroni correction for SCL scales and CTQ: 0.05/72 = 0.00042*; and IRI: 0.05/48 = 0.0010*; Therefore, only correlations with p < 0.001 were considered as significant. (DOCX) [file pone.0248514.s003.docx]

**S3 Table. Spearman’s rho correlation coefficients (*r* (*p*)) calculated between psychopathological symptoms (SCL-90-R), childhood trauma (CTQ) and empathy (IRI).**

|  |  | **CTQ Total score** | **CTQ**  **Emotional abuse** | **CTQ Physical abuse** | **CTQ Sexual abuse** | **CTQ**  **Emotional neglect** | **CTQ**  **Physical neglect** | **IRI Perspec-tive**  **taking** | **IRI Fantasy** | **IRI Empathic**  **concern** | **IRI Personal distress** | |  |
| --- | --- | --- | --- | --- | --- | --- | --- | --- | --- | --- | --- | --- | --- |
| **SCL-90-R** | | | | | | | | | | | |  | |
|  | Somatization | **0.428*** **(<0.001)** | **0.387*** **(<0.001)** | **0.322**  **(0.003)** | **0.308 (0.004)** | **0.375**  **(<0.001)** | **0.449**  **(<0.001)** | **-0.212**  **(0.050)** | 0.020 (0.854) | 0.124 (0.255) | **0.430*** **(<0.001)** | |  |
|  | Obsessive-compulsiveness | **0.589* (<0.001)** | **0.595*** **(<0.001)** | **0.529*** **(<0.001)** | **0.420*** **(<0.001)** | **0.524*** **(<0.001)** | **0.504*** **(<0.001)** | **-0.304**  **(0.004)** | -0.040 (0.715) | 0.017 (0.875) | **0.694*** **(<0.001)** | |  |
|  | Social insecurity | **0.569*** **(<0.001)** | **0.598*** **(<0.001)** | **0.381***  **(<0.001)** | **0.406*** **(<0.001)** | **0.538*** **(<0.001)** | **0.465*** **(<0.001)** | **-0.368***  **(<0.001)** | -0.056 (0.609) | -0.013 (0.907) | **0.682*** **(<0.001)** | |  |
|  | Depression | **0.633*** **(<0.001)** | **0.625*** **(<0.001)** | **0.466**  **(0.001)** | **0.421**  **(0.001)** | **0.606*** **(<0.001)** | **0.572*** **(<0.001)** | **-0.420***  **(<0.001)** | -0.044 (0.689) | -0.013 (0.906) | **0.711*** **(<0.001)** | |  |
|  | Anxiety | **0.572*** **(<0.001)** | **0.591*** **(<0.001)** | **0.484*** **(<0.001)** | **0.476 (0.001)** | **0.508*** **(<0.001)** | **0.494*** **(<0.001)** | **-0.337**  **(0.002)** | -0.029 (0.793) | 0.019 (0.862) | **0.608*** **(<0.001)** | |  |
|  | Aggression | **0.530*** **(<0.001)** | **0.547*** **(<0.001)** | **0.471 (0.001)** | **0.479* (<0.001)** | **0.443*** **(<0.001)** | **0.4430*** **(<0.001)** | **-0.363**  **(0.001)** | -0.014 (0.895) | -0.023 (0.836) | **0.689*** **(<0.001)** | |  |
|  | Phobic anxiety | **0.430*** **(<0.001)** | **0.403*** **(<0.001)** | **0.345**  **(0.001)** | **0.340 (0.001)** | **0.397*** **(<0.001)** | **0.367*** **(0.001)** | **-0.303**  **(0.005)** | 0.018 (0.871 | 0.075 (0.493) | **0.539*** **(<0.001)** | |  |
|  | Paranoid thinking | **0.475*** **(<0.001)** | **0.452*** **(<0.001)** | **0.345* (0.001)** | **0.361 (0.001)** | **0.398*** **(<0.001)** | **0.373*** **(<0.001)** | **-0.250**  **(0.020)** | 0.035 (0.748) | 0.013 (0.906) | **0.542*** **(<0.001)** | |  |
|  | Psychoticism | **0.545*** **(<0.001)** | **0.556*** **(<0.001)** | **0.439 (0.001)** | **0.441*** **(<0.001)** | **0.512*** **(<0.001)** | **0.469*** **(<0.001)** | **-0.215**  **(0.047)** | 0.012 (0.915) | 0.023 (0.830) | **0.525*** **(<0.001)** | |  |
|  | GSI | **0.537*** **(<0.001)** | **0.554*** **(<0.001)** | **0.413*** **(<0.001)** | **0.373*** **(0.002)** | **0.516*** **(<0.001)** | **0.450*** **(<0.001)** | **-0.366***  **(0.001)** | -0.089 (0.416) | 0.036 (0.744) | **0.641*** **(<0.001)** | |  |
|  | PST | **0.575*** **(<0.001)** | **0.573*** **(<0.001)** | **0.467 (0.001)** | **0.391 (0.003)** | **0.535*** **(<0.001)** | **0.489*** **(<0.001)** | **-0.245**  **(0.023)** | -0.060 (0.582) | 0.022 (0.837) | **0.565*** **(<0.001)** | |  |
|  | PSDI | **0.526*** **(<0.001)** | **0.497*** **(<0.001)** | **0.339**  **(0.001)** | **0.361 (0.001)** | **0.493*** **(<0.001)** | **0.439*** **(<0.001)** | **-0.407***  **(<0.001)** | -0.034 (0.758) | 0.042 (0.703) | **0.617*** **(<0.001)** | |  |

Bonferroni correction for SCL scales and CTQ: 0.05/ 72 = 0.00042*; and IRI: 0.05/ 48 = 0.0010*; Therefore, only correlations with *p* < 0.001 were considered as significant.
