# Supplementary material for: Association between childhood maltreatment, psychopathology and DNA methylation of genes involved in stress regulation: Evidence from a study in Borderline Personality Disorder
Source: PLoS One. 2021 Mar 11;16(3):e0248514. doi: 10.1371/journal.pone.0248514 (PMC7951851; doi:10.1371/journal.pone.0248514)
Supplement: S4 Table — Bonferroni correction for IRI scales: 0.05/24 = 0.0021*. (DOCX) [file pone.0248514.s004.docx]

**S4 Table**. **Correlations (Spearman (*r* (*p*)) between childhood trauma scales (CTQ) and empathy (IRI).**

|  |  | **IRI Perspective**  **taking** | **IRI Fantasy** | **IRI Empathic**  **concern** | **IRI Personal distress** |
| --- | --- | --- | --- | --- | --- |
| Childhood Trauma Questionnaire | | |  |  |  |
|  | **Emotional abuse** | **-0.347*** **(0.001)** | -0.077 (0.471) | -0.081 (0.450) | **0.596* (<0.001)** |
|  | **Physical abuse** | **-0.209 (0.049)** | -0.118 (0.271) | -0.130 (0.226) | **0.427* (<0.001)** |
|  | **Sexual abuse** | **-0.268 (0.011)** | -0.061 (0.569) | -0.007 (0.949) | **0.407* (<0.001)** |
|  | **Emotional neglect** | **-0.256 (0.015)** | -0.090 (0.399) | -0.143 (0.181) | **0.528* (<0.001)** |
|  | **Physical neglect** | -0.207 (0.052) | -0.066 (0.542) | -0.027 (0.802) | **0.4420* (<0.001)** |
|  | **Total score** | **-0.269 (0.011)** | -0.080 (0.428) | -0.033 (0.078) | **0.586* (<0.001)** |

Bonferroni correction for IRI scales: 0.05/ 24 = 0.0021*.
