# Supplementary material for: Association between childhood maltreatment, psychopathology and DNA methylation of genes involved in stress regulation: Evidence from a study in Borderline Personality Disorder
Source: PLoS One. 2021 Mar 11;16(3):e0248514. doi: 10.1371/journal.pone.0248514 (PMC7951851; doi:10.1371/journal.pone.0248514)
Supplement: S5 Table — Significant correlations are printed in bold, however these correlation did not survive correction due to multiple testing. Bonferroni correction for IRI for each region/bin: 0.05/6 = 0.0083*. (DOCX) [file pone.0248514.s005.docx]

**S5 Table**. **Correlations (Spearman (*r* (*p*)) between childhood trauma scales (CTQ) and FKBP5 (bin 1 mean, bin 2 mean, mean bin 1 and 2) and NR3C1 mean methylation for patients with BPD and HC (n = 88). Significant correlations are printed in bold, however, these correlations did not survive correction for multiple testing.**

| Childhood Trauma Questionnaire | **FKBP5 bin 1** | **FKBP5 bin 2** | **FKBP5 mean** | **NR3C1 mean** |
| --- | --- | --- | --- | --- |
| **Emotional abuse** | 0.023 (0.830) | -0.055 (0.609) | -0.041 (0.705) | **-0.267 (0.011)** |
| **Physical abuse** | -0.024 (0.824) | -0.073 (0.501) | 0.038 (0.722) | **-0.251 (0.018)** |
| **Sexual abuse** | -0.136 (0.207) | 0.108 (0.318) | -0.002 (0.988) | -0.128 (0.232) |
| **Emotional neglect** | 0.045 (0.677) | 0.057 (0.601) | 0.049 (0.650) | -0.200 (0.060) |
| **Physical neglect** | 0.144 (0.182) | 0.139 (0.197) | 0.144 (0.180) | **-0.233 (0.028)** |
| **Total score** | 0.021 (0.848) | 0.110 (0.306) | 0.074 (0.491) | **-0.226 (0.033)** |

Bonferroni correction for IRI for each region/bin: 0.05/ 6 = 0.0083*.
