# Supplementary material for: Association between childhood maltreatment, psychopathology and DNA methylation of genes involved in stress regulation: Evidence from a study in Borderline Personality Disorder
Source: PLoS One. 2021 Mar 11;16(3):e0248514. doi: 10.1371/journal.pone.0248514 (PMC7951851; doi:10.1371/journal.pone.0248514)
Supplement: S6 Table — (DOCX) [file pone.0248514.s006.docx]

**S6 Table**. **Correlations between IRI scales.**

|  | **Fantasy** | **Empathic concern** | **Personal distress** |
| --- | --- | --- | --- |
| **Perspective taking** | 0.358 (0.001) | 0.353 (0.001) | **-0.394* (<0.001**) |
| **Fantasy** |  | **0.372* (<0.001)** | 0.014 (0.895) |
| **Empathic concern** |  |  | 0.162 (0.129) |
